# Single-epitope recognition imaging of native chromatin

**DOI:** 10.1186/1756-8935-1-10

**Published:** 2008-12-17

**Authors:** Hongda Wang, Yamini Dalal, Steven Henikoff, Stuart Lindsay

**Affiliations:** 1 Biodesign Institute, Arizona State University, Tempe, AZ 85287, USA; 2 State Key Laboratory of Electroanalytical Chemistry, Changchun Institute of Applied Chemistry, Chinese Academy of Sciences, Changchun 130022, PR China; 3 Basic Sciences Division, Fred Hutchinson Cancer Research Center, Seattle, WA 98109, USA; 4 National Cancer Institute, Laboratory of Receptor Biology and Gene Expression, National Institutes of Health, Bethesda, MD 20892, USA; 5 Howard Hughes Medical Institute, Jones Bridge Road, Chevy Chase, MD 20815-6789, USA

## Abstract

**Background:**

Direct visualization of chromatin has the potential to provide important insights into epigenetic processes. In particular, atomic force microscopy (AFM) can visualize single nucleosomes under physiological ionic conditions. However, AFM has mostly been applied to chromatin that has been reconstituted *in vitro*, and its potential as a tool for the dissection of native nucleosomes has not been explored. Recently we applied AFM to native Drosophila chromatin containing the centromere-specific histone 3 (CenH3), showing that it is greatly enriched in smaller particles. Taken together with biochemical analyses of CenH3 nucleosomes, we propose that centromeric nucleosomes are hemisomes, with one turn of DNA wrapped around a particle consisting of one molecule each of centromere-specific CenH3, H4, H2A and H2B.

**Results:**

Here we apply a recognition mode of AFM imaging to directly identify CenH3 within histone core particles released from native centromeric chromatin. More than 90% of these particles were found to be tetrameric in height. The specificity of recognition was confirmed by blocking with a CenH3 peptide, and the strength of the interaction was quantified by force measurements. These results imply that the particles imaged by AFM are indeed mature CenH3-containing hemisomes.

**Conclusion:**

Efficient and highly specific recognition of CenH3 in histone core particles isolated from native centromeric chromatin demonstrates that tetramers are the predominant form of centromeric nucleosomes in mature tetramers. Our findings provide proof of principle that this approach can yield insights into chromatin biology using direct and rapid detection of native nucleosomes in physiological salt concentrations.

## Background

Eukaryotic genomes are packaged with octameric protein particles, consisting of two copies each of histones H2A, H2B, H3 and H4, which wrap nearly two turns of DNA to form nucleosomes [1]. Since the discovery of nucleosomes in the early 1970s, a variety of techniques have been applied to their study. However, both ultrastructural technologies (e.g. crystallography and electron microscopy) and biochemical analyses (e.g. nuclease assays and sedimentation) have been limited in their scope, because they cannot simultaneously assay structure and dynamics. In recent years, progress has been made in applying new technologies that have the potential to bridge the gap between static ultrastructural features and dynamic physiological processes in the study of chromatin. These technologies, which include scanning confocal fluorescence microscopy [2], molecular tweezers [3,4] and atomic force microscopy (AFM) [5,6], have provided remarkable insights into the behavior of individual nucleosomes. The combination of single-molecule resolution, solution biochemistry and observation of native macromolecular complexes has made AFM especially attractive for studying nucleosomes.

A fundamental level of distinction between nucleosome types is provided by the incorporation of alternative variants of histones H2A and H3 [7]. At chromosomal sites for spindle fiber attachment at mitosis, histone H3 is replaced with a centromere-specific variant (CenH3, CENP-A in humans) to form the specialized nucleosomes that comprise centromeric chromatin [8]. CenH3s in a variety of eukaryotes are found to be absolutely essential for the organization of the kinetochore, which is the only chromosomal structure required for mitosis and meiosis [9]. Therefore, CenH3 nucleosomes are thought to provide the molecular foundation for assembly of the kinetochore at mitosis [10].

Until recently, the structure of CenH3 nucleosomes was presumed to be the same as that for canonical H3 nucleosomes. However, our recent study showed that CenH3 nucleosomes consist of half the DNA and protein of octameric nucleosomes, with one molecule each of CenH3, H4, H2A and H2B [11]. In that study, we applied AFM to complement detailed biochemical characterization, and found that CenH3 chromatin consists of particles that are half the height of bulk octameric nucleosomes. However, it has been argued that the tetrameric particles that we observed might be non-nucleosomal intermediates in the assembly of CenH3 chromatin [12]. Given the novelty of tetrameric nucleosomes in eukaryotic biology [13], and the potential for controversy [14], it is important to directly test alternative interpretations of our observations.

To address the possibility that we have identified an immature intermediate in CenH3 nucleosome assembly as opposed to a mature form, we adapted the purification of the CenH3 nucleosomes to include a DNA-binding step, so that only stable nucleosomes are purified rather than soluble assembly intermediates. In addition, we have applied recognition imaging, wherein an anti-CenH3 antibody is covalently coupled to the AFM tip. Recognition imaging provides highly efficient and specific identification of epitopes within nucleosomes [15]. This strategy has allowed us to show directly that by far the most predominant form of CenH3 particles isolated from chromatin is tetrameric in height, which is inconsistent with them being assembly intermediates. Our application of single-epitope recognition imaging to centromeric nucleosomes also illustrates the potential of recognition imaging by AFM for analyzing minute amounts of specialized chromatin fractions isolated from the nucleus.

## Results and discussion

### Recognition imaging of centromeric nucleosomes

**Figure 1:**
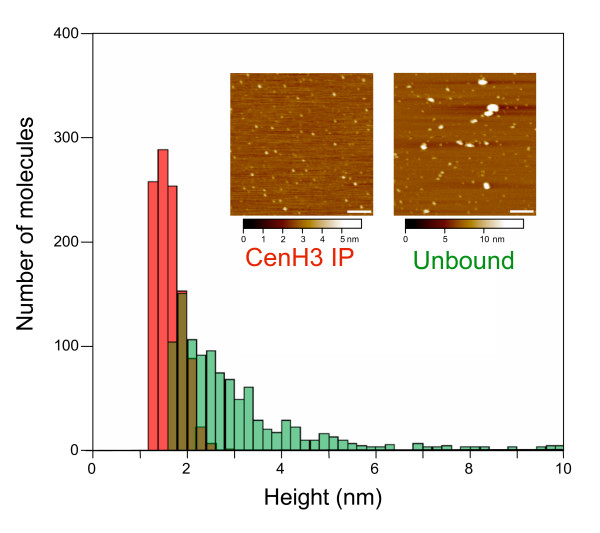
**Native CenH3 core particle complexes are tetrameric under physiological salt concentrations**. Hydroxylapatite-bound chromatin was purified from interphase cells to enrich for histone core particle complexes immunoprecipitated using an anti-CenH3 antibody and visualized by atomic force microscopy. The unbound material contains predominantly canonical histone core particles and provided a control. Only particles more than 1.65 nm high and 6 nm in radius in the samples were measured to exclude small non-nucleosomal particles and debris. Heights were measured for an equal number of particles for CenH3 (red) and unbound (green) particles. Sample images are shown as insets, where the bar is 100 nm, and the *Z *range is shown at the bottom of the images.

To distinguish between mature nucleosomes and immature intermediates, we immobilized glutaraldehyde-fixed chromatin on hydroxylapatite, washed in 0.35 M salt to remove non-histone proteins, and then eluted intact histone core particles with 2 M salt. CenH3 particles were enriched from this soluble material by immunoprecipitation using a CenH3 antibody. We first characterized the homogeneity of these preparations using conventional AFM. Figure 1 shows height measurements for a sample of CenH3-immunoprecipitated histone complexes together with a set of height measurements for canonical histone complexes, both imaged in parallel at physiological salt concentrations. As we had previously shown for CenH3 nucleosomes [11], CenH3 core particles display a tight distribution between 1 and 2 nm, consistent with their proposed hemisomal organization. In contrast, unbound core particles display a broad distribution of heights that are on average about twice as tall as CenH3 core particles, consistent with their known octameric organization [6].

**Figure 2:**
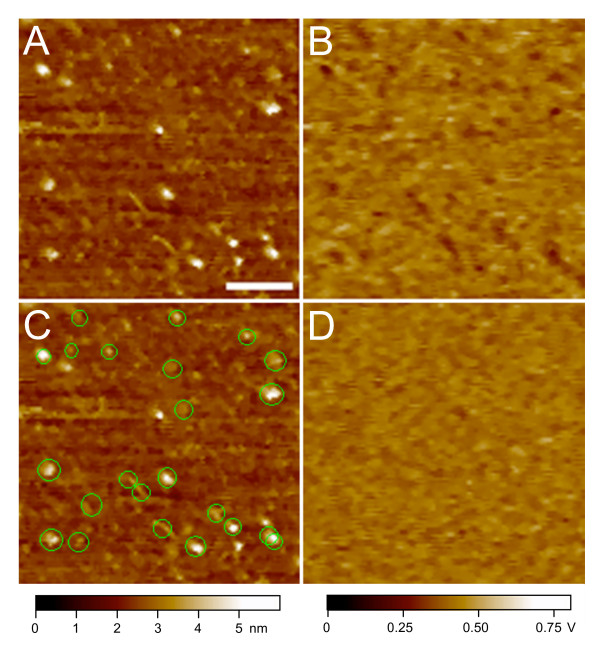
**Recognition imaging of native CenH3 core particle complexes**. CenH3 core particles obtained by hydroxylapatite enrichment of chromatin followed by immunoprecipitation were visualized by an anti-CenH3 atomic force microscopy tip (A). The recognition signal is detected as dark spots in the recognition image (B). In the overlay shown in (C), the recognition signal is marked with green dots for visual clarity using custom software. No recognition signal is seen after incubation with a peptide corresponding to the CenH3 epitope (D). The bar is 100 nm and the *Z *range and volt scale are shown at the bottom of the images.

In recognition imaging, the AFM tip is tagged with an anti-CenH3 antibody, and small differences in the probe deflection signal are indicative of an antibody-epitope binding event [16]. A map of these recognition signals is generated together with the topographic image, with recognition events appearing as 'dark spots' on a second screen. Figure 2A shows the topographic image of CenH3 histone core particles deposited on a mica surface and scanned using the anti-CenH3 antibody tip. Recognition images were obtained simultaneously. Figure 2B shows the recognition image, where the anti-CenH3 antibody has interacted with its epitope. An overlay of the two images, with the recognition spots circled in green, demonstrates excellent correspondence between the CenH3 nucleosomes and epitope by the tethered tip (Figure 2C). This nearly one-to-one correspondence between the antibody-tethered tip and the single CenH3 epitope per core particle complex demonstrates the exquisite sensitivity of direct recognition imaging. In a survey of 339 total particles measured in our experiments, 313 (92%) were positively identified as CenH3. The small remainder that could not be identified might reflect contamination from H3-containing particles [11], or concealed or degraded epitopes within the CenH3 N-terminal tail. The robust detection of more than 90% of the particles is highly specific, because blocking with the CenH3 peptide to which the antibody was raised resulted in a complete reversal of recognition (Figure 2D). Furthermore, the use of hydroxylapatite purification prior to immunoprecipitation implies that robust detection of CenH3 in tetramer-height particles applies to the entire population of nucleosomes containing the CenH3 epitope.

**Figure 3:**
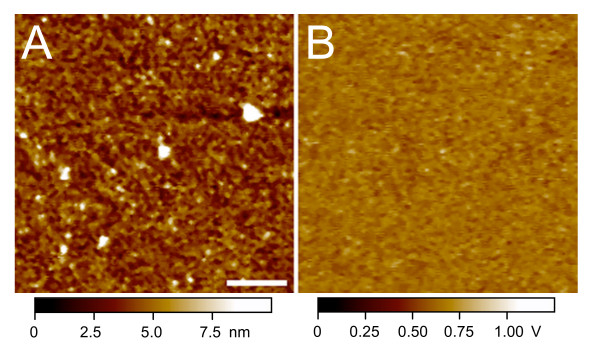
**Recognition imaging of unbound canonical core particle complexes**. The unbound canonical core particle fraction obtained from the CenH3 immunoprecipitation step was imaged using an anti-CenH3 tip as in Figure 2. (A) Topographic image. (B) Recognition image. No recognition signal is detected. The bar is 100 nm. The *Z *range is shown at the bottom of the images.

To rule out the possibility that the antibody-tethered tip is simply sticking non-specifically to chromatin projections off the mica surface, we tested canonical histone core particles from the same experiment in parallel. This sample provided a stringent control, because bulk core particles are octameric and, therefore, on average twice as tall as CenH3 particles ([11] and reproduced here). Even so, no recognition signal was obtained when bulk core particles were imaged with the CenH3 antibody (Figure 3).

### Force analysis of tip-sample dissociation

**Figure 4:**
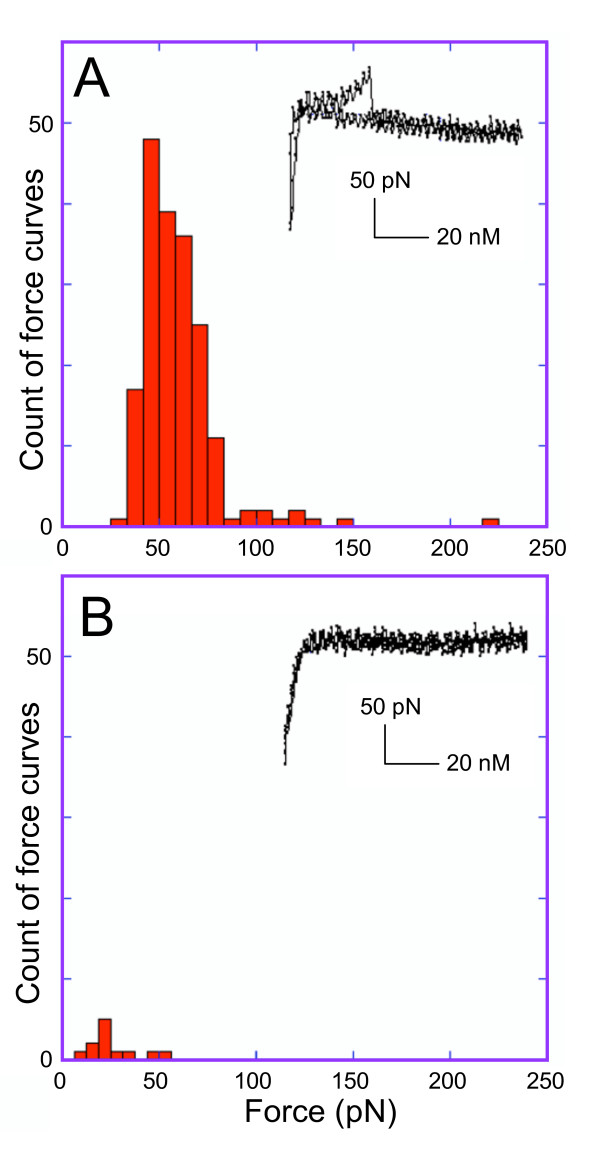
**Force measurements of unbinding events for an anti-CenH3 tip**. Histograms for the distribution of force measurements for (A) 300 CenH3 particle force curves and (B) 12 bulk particle force curves. Typical force curves for the two samples are depicted in the insets.

A quantitative measure of specificity of the interaction between the anti-CenH3 tip and its epitope in native complexes can be obtained by measuring the unbinding force exerted by the tip required to break the interaction between the anti-CenH3 antibody and its CenH3 target. Therefore, the higher the force, the greater the specificity of interaction relative to control. The value of the unbinding force is 63.1 ± 20.8 pN (standard error) measured over 300 CenH3 force curves (Figure 4A). These values are in accordance with previously reported values for antibody unbinding forces that typically range between 40 and 100 pN [16]. In contrast, the unbinding force for the bulk nucleosomal control is almost undetectable over background measured over 300 force curves (Figure 4B). The corresponding force curves are depicted in the insets of Figure 4. These curves reflect a gradual increase in tension until abrupt release of binding occurs for CenH3 nucleosomes but not for control nucleosomes. We conclude that the interaction between the anti-CenH3 antibody tip and CenH3 nucleosomes is highly specific.

Our application of recognition imaging to CenH3 nucleosomes also serves to highlight the exquisite sensitivity of this technique, wherein each particle detected represents the signal from a single CenH3 protein epitope that is present at the end of the 125-aa N-terminal tail. Single-epitope recognition also suggests broader applications of AFM to native chromatin. There has been an explosion of interest in the distribution of histone modifications and variants in chromatin to understand epigenetic regulation [17], and as a result, there are now dozens of excellent commercially available antibodies against a wide variety of histone modifications. The methods that we have described here for CenH3 nucleosomes should be broadly applicable to any histone variant or modification, especially in cases where only small amounts of native material are available. Therefore, AFM recognition imaging has the potential to provide single-molecule maps of a wide variety of epigenetic features for any chromatin fraction that can be isolated.

## Conclusion

Direct measurement of single CenH3 epitopes in the tetramer-height particles released from native chromatin reveals that more than 90% contain CenH3. Insofar as an assembly intermediate would, by definition, comprise only a minor subset of the entire population, we conclude that CenH3 hemisomes represent the mature form of centromeric chromatin. Thus, the high sensitivity and specificity of single-epitope recognition imaging has made it possible to address a key issue in chromatin biology. With efficient single-epitope detection of native macromolecular complexes, AFM with recognition imaging should be generally applicable to the detection of epitopes in biological samples that are available in only limited amounts.

## Methods

### Biochemical purification of native histone core particles

**Figure 5:**
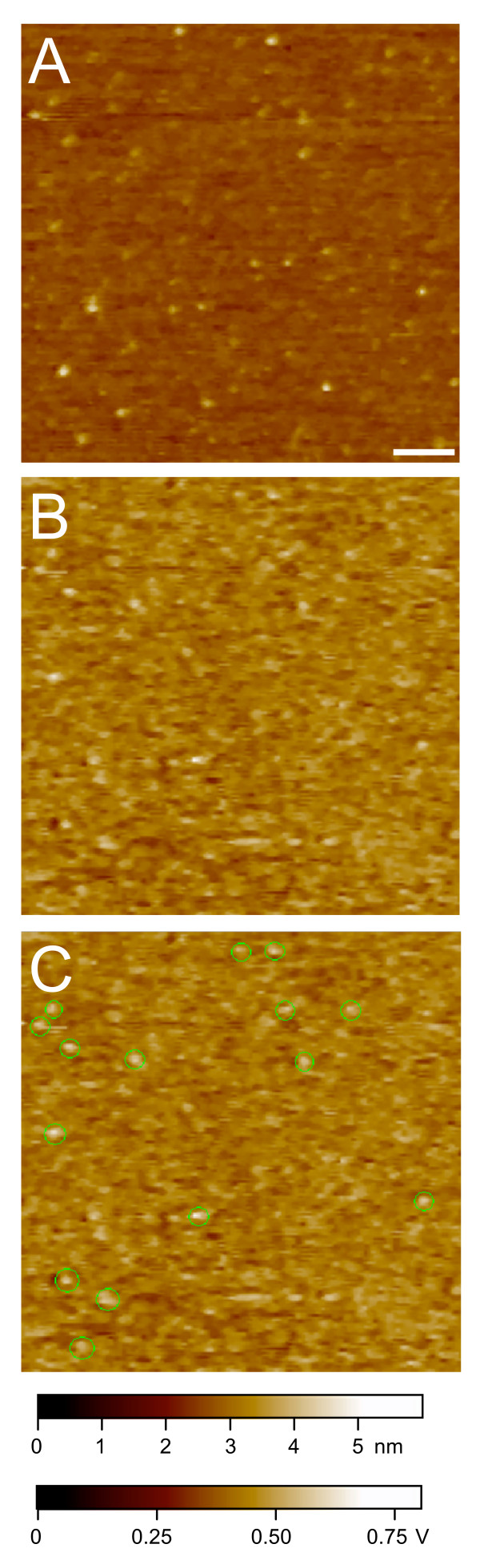
**High recognition signal background when CenH3 peptide is not removed by dialysis**. (A) Topographic image of a CenH3 immunoprecipitation sample without dialysis. Scale bar is 100 nm. (B) The corresponding recognition image. (C) Same as (B), where the expected recognition sites are marked by green circles.

Drosophila S2 cells (100 ml) were grown to mid-log phase. Cells were collected by centrifugation and processed as described previously [11] to obtain purified CenH3 and bulk nucleosomal particles with the following adaptations. Chromatin was released from interphase nuclei in S2 cells by micrococcal nuclease (MNase) digestion, and treated *in situ *within intact nuclei with 0.05% glutaraldehyde to preserve histone core particle organization. The nuclei were subsequently bound to hydroxylapatite in 1× phosphate buffered saline (PBS) supplemented with 0.35 M NaCl, 0.2 mM ethylenediaminetetraacetic acid (EDTA) and 0.5 mM phenylmethanesulphonyl fluoride (PMSF) for several hours with slow stirring to create a slurry [18]. Hydroxylapatite specifically binds DNA, therefore only DNA-associated histones are enriched during this process. Hydroxylapatite-bound chromatin was collected by low-speed centrifugation and washed twice with 0.35 M NaCl-PBS to remove nuclear proteins that are not bound to DNA, most non-histone chromosomal proteins, and incompletely assembled nucleosomes. The hydroxylapatite-bound chromatin was then incubated with 2 × 20 ml volumes of ice-cold 2 M NaCl-PBS supplemented to 0.5 mM PMSF and 0.2 mM EDTA with slow stirring at 4°C. Under these conditions, intact histone core particles completely dissociate from DNA [18]. The core particles were concentrated to 2 ml using an Amicon pressure cell. The salt was dialyzed down to 0.35 M NaCl-PBS supplemented with 0.5 mM PMSF. Centromeric particles were enriched from this material by immunoprecipitation using a 1:1000 dilution of CenH3 antibody overnight in an end-over-end rotator. The unbound fraction containing mostly canonical histone core particles was saved as the control. Immunoprecipitated samples were washed once in 0.35 M NaCl-PBS, once in 0.15 M NaCl-PBS, and then eluted from the beads by competition with 1 mg ml^-1 ^CenH3-specific peptide for 2–4 hours. Eluted particles were dialyzed as 100 μl droplets on 0.025 μm pore dialysis disks (Millipore) against 50 ml of 1 × PBS buffer for 1 hour at room temperature to remove unbound peptide, diluted between 1:10 and 1:100 and imaged by AFM directly in 1 × PBS within 48 hours of preparation. The dialysis step eliminated a high recognition signal background that was seen without dialysis (Figure 5), which indicates that the recognition signals obtained for samples used in this study did not result from residual peptide sticking to the mica surface. The affinity-purified CenH3 antibody had been raised to a peptide epitope present at end of the 125-aa N-terminal tail of Drosophila CenH3 (Cid [19]). This epitope is not thought to participate in intra-nucleosome histone:histone interactions, and in the absence of DNA is likely to remain accessible to the imaging tip during recognition events.

### APTES-mica preparation

A desiccator was purged with argon for 2 minutes and 30 μl of APTES (99% 3-aminopropyl triethoxysilane, Sigma-Aldrich, St. Louis, MO) placed into a small container at the bottom of the desiccator. Ten microliters of *N*,*N*-diisopropylethylamine (99%, distilled, Sigma-Aldrich) was placed into another small container, and the desiccator purged with argon for a further 2 minutes. Mica sheets were stripped on one side until smooth and immediately placed into the desiccator. The desiccator was purged for another 3 minutes and then sealed off, leaving the mica exposed to APTES vapor for 1 hour. After this exposure, the APTES was removed, the desiccator purged, and the APTES-mica stored in the sealed desiccator until needed.

### Preparing samples for AFM imaging

Two hundred microliters of a 2 μM glutaradehyde (grade I, Sigma-Aldrich) solution in water was added via a pipette onto APTES-mica immediately upon removal from the storage desiccator and incubated for 10 minutes [20]. The surface was rinsed with water from a Nanopure ultrapure water system, and 60 μl core particle solution (about 0.2 μg core particles per milliliter in PBS buffer) was added via a pipette onto the treated surface and allowed to incubate for 30 minutes. The surface was then rinsed again with PBS buffer (100 mM NaCl, 50 mM Na-phosphate, pH 7.5). The prepared sample was mounted into the scanning probing microscopy (SPM) liquid flow cell and imaged immediately.

### Functionalizing AFM tips

Silicon-nitride cantilever tips (Microlever, Veeco, Santa Barbara, CA, coated for MacMode AFM by Agilent Technologies, Chandler, AZ) for recognition imaging were used as described [16]. Briefly, anti-CenH3 antibody was reacted with N-Succinimidyl 3-(acetylthio)propionate (SATP, Sigma inc.) and purified in a PD-10 column (Amersham Pharmacia Biotech). The cantilevers were cleaned in a ultraviolet (UV) cleaner, vapor-treated with APTES and reacted with polyethylene glycol (PEG) crosslinker using triethylamine and CHCl_3_. The SATP-labeled antibodies were then bound to the PEG crosslinkers with NH_2_OH (Sigma) in NaCl/Phosphate buffer. The tips were then rinsed in PBS buffer and stored at 4°C until use.

### Imaging of native core particles

Conventional imaging was performed on a Pico I MacMode AFM (Agilent Technologies, Chandler, AZ) with an amplitude setting between 2.0 and 2.5 V. Recognition imaging was performed on a Pico I AFM with a Picotrec recognition imaging attachment (Agilent Technologies) with an amplitude setting of about 14–16 nm. AFM engagement was performed at 30% amplitude reduction. The images were taken in PBS buffer. Peptide blocking experiments (Figure 2D) were performed by adding 200 μl CenH3 peptide (30 μg ml^-1^) into the liquid cell (diameter 1.2 cm, height 0.5 cm) during imaging of samples resulting in an effective peptide concentration of 10 μg cm^-2^.

### Data analysis

Recognition imaging was analyzed using custom software [21,22]. This program compiles histograms of the pixel intensity distribution from background regions containing no features, and compares them with regions containing visible recognition spots. Recognition events give rise to a second peak in the intensity distribution and these were clearly separated from the background by selecting a cut-off of 75% of the background intensity (recognition spots correspond to a decrease in the signal). Particle heights were measured using FemtoScan (Advanced Technologies Center). A maximum height was taken as the peak height relative to the local background. Only particles with an apparent diameter of more than 12 nm were counted. The true diameter of histone core particle ranges from 8 to 10 nm in the absence of DNA [23], which produces features from 15 to 25 nm in diameter in the AFM image owing to the limited resolution of the probe.

## Competing interests

The authors declare that they have no competing interests.

## Authors' contributions

HW, YD and SL conceived and designed the experiments, YD prepared the native core particles and HW performed the AFM experiments. HW, YD, SH and SL analyzed the data, and YD, SH and HW wrote the paper.
